# The Biography of Ephraim McDowell, M. D.

**Published:** 1890-12

**Authors:** 


THE BIOGRAPHY OF EPHRAIM McDOWELL, M. D., "The Father of Ovariotomy." By his granddaughter, MARY YOUNG RIDENBAUGH. Together with valuable scientific treatises and articles relating to Ovariotomy; also eulogistic letters from eminent members of the medical profession in Europe and America. Small octavo, full Russia, gilt; pp. xvi.—538. New York: Charles L. Webster & Company. 1890. William J. Dornan, Philadelphia, Printer.

The memory of Ephraim McDowell is very dear to every American physician, and ought to be treasured by every woman in the land.

When we reflect that all the splendid record that abdominal surgery has ever made, has been built around that heroic feat performed by the stalwart Kentuckian in his humble home in Danville, it cannot be asserted that we are extravagant in our desire to have McDowell's name revered by all American physicians.

The work before us is a tribute of affection from a near kinswoman of the great McDowell, and will be read with interest by all who are fortunate enough to possess it or obtain access to its pages.

It is a beautifully printed volume, on excellent paper, and is bound in exquisite taste. It will at once furnish ready reference not only to McDowell's own work, but to that of many who have succeeded him in enlarging the field that he first cultivated, and ought to be found in the library of every physician.

It can be obtained in three different styles of binding upon application to the author, Mrs. Mary Young Ridenbaugh, P. O. Drawer 372, Philadelphia, Pa.

